# Central insulin action induces activation of paraventricular oxytocin neurons to release oxytocin into circulation

**DOI:** 10.1038/s41598-018-28816-w

**Published:** 2018-07-10

**Authors:** Boyang Zhang, Masanori Nakata, Jun Nakae, Wataru Ogawa, Toshihiko Yada

**Affiliations:** 1 Department of Physiology, Division of Integrative Physiology, Faculty of Medicine, Jichi Medical University, 3311-1 Yakushiji, Shimotsuke, Tochigi 329-0498 Japan; 2 Department of Physiology, Faculty of Medicine, Wakayama Medical University School of Medicine, 641-8509, Kimiidera 811-1, Wakayama, Wakayama Japan; 3 Frontier Medicine on Metabolic Syndrome, Division of Endocrinology, Metabolism and Nephrology, Department of Internal Medicine, Keio University School of Medicine, Tokyo, Japan; 4 Department of Internal Medicine, Division of Diabetes and Endocrinology, Kobe University Graduate School of Medicine, Kobe, Japan; 5 Kansai Electric Power Medical Research Institute, 1-5-6 Minatojimaminamimachi, Chuou-ku, Kobe 650-0047 Japan

## Abstract

Oxytocin neurons in the paraventricular nucleus (PVN) of hypothalamus regulate energy metabolism and reproduction. Plasma oxytocin concentration is reduced in obese subjects with insulin resistance. These findings prompted us to hypothesize that insulin serves to promote oxytocin release. This study examined whether insulin activates oxytocin neurons in the PVN, and explored the underlying signaling. We generated the mice deficient of 3-phosphoinositide-dependent protein kinase-1 (PDK1), a major signaling molecule particularly for insulin, specifically in oxytocin neurons (*Oxy Pdk1* KO). Insulin increased cytosolic calcium concentration ([Ca^2+^]_i_) in oxytocin neurons with larger (≧25 μm) and smaller (<25 μm) diameters isolated from PVN in C57BL/6 mice. In PDK1 *Oxy Pdk1* KO mice, in contrast, this effect of insulin to increase [Ca^2+^]_i_ was markedly diminished in the larger-sized oxytocin neurons, while it was intact in the smaller-sized oxytocin neurons. Furthermore, intracerebroventricular insulin administration induced oxytocin release into plasma in *Oxy Cre* but not *Oxy Pdk1* KO mice. These results demonstrate that insulin PDK1-dependently preferentially activates PVN magnocellular oxytocin neurons to release oxytocin into circulation, possibly serving as a mechanism for the interaction between metabolism and perinatal functions.

## Introduction

Insulin has various effects in multiple tissues, including stimulation of glucose uptake in skeletal muscle and adipose tissue and suppression of gluconeogenesis and glycogenolysis in the liver, thereby lowering the blood glucose level. In addition to the peripheral action, insulin is known to lower blood glucose via central action^1,2^. Insulin receptor is widely expressed in the brain, including hypothalamus, olfactory bulb, hippocampus, cerebral cortex and cerebellum^3^. A knockout of insulin receptor in neuronal tissue (NIRKO) showed increases in body weight, white adipose tissue, and serum triglycerides, and these changes were more pronounced in females^4^. Furthermore, NIRKO mice of both sexes showed reduced fertility. Diabetes mellitus is known to increase the frequency of maternal complications during perinatal periods in pregnancies. These findings have demonstrated the importance of insulin in maintaining reproduction and perinatal functions. However, the effect of central insulin on the hormone that regulates reproduction and perinatal functions remains unclear.

Oxytocin, a neuropeptide with 9 amino acid residues, is produced by two types of oxytocin neurons in the hypothalamus; the magnocellular neurons in the paraventricular nucleus (PVN) and supraoptic nucleus (SON), and the parvocellular neurons in PVN. The parvocellular oxytocin neurons in PVN project to the hypothalamus, limbic system and brain stem, and are involved in sexual behavior, social adaptive behavior, feeding behavior, learning and memory^5–10^. The magnocellular oxytocin neurons release oxytocin into the blood vessels from their axon terminals in the posterior pituitary gland, and thereby elicit uterine contraction and milk ejection, contributing to reproduction and perinatal functions. Recently, oxytocin has been shown to play an important role in maintenance of metabolism and energy balance^11–15^. Inversely, decreased plasma level of oxytocin in obese patients was reported^16^. These findings prompted us to hypothesize that insulin serves to promote oxytocin release. In this study, we aimed to clarify whether insulin activates oxytocin neurons in the PVN and, if so, to explore the underlying signaling pathway.

## Methods

### Animals

Male C57BL/6 mice (SLC, Hamamatsu, Japan) were single-housed for *in vivo* experiments and group-housed for Ca^2+^ imaging under a 12-h light/dark cycle condition (7:30 light on). Food (Standard animal chow CE-2; CLEA, Osaka, Japan) and water were available *ad libitum* except particular experiments performed under fasted conditions.

We generated oxytocin neuron-specific 3-phosphoinositide-dependent protein kinase-1 (PDK1) knockout (*Oxy Pdk1 KO*) mice by mating Oxytoin-Ires-Cre knock-in mice (*Oxy Cre*) (a generous gift from Dr. Bradford B Lowel, Beth Israel Deaconess Medical Center/Harvard Medical School) with *Pdk1*-floxed mouse with loxP sites flanking exons 3 and 4^2,15^. All mice were genotyped by polymerase chain reaction (PCR) amplification of genomic DNA isolated from tail tips. Both *Oxy Cre* mice and *Oxy Pdk1KO* mice were maintained in the same conditions as for C57BL/6 mice. Male mice were used for the experiment. All animal procedures were conducted in compliance with protocols approved by Jichi Medical University Animal Care and Use Committee.

### Intracerebroventricular (ICV) injection

Mice aged 9 weeks were anesthetized by intraperitoneal (ip) injection of pentobarbitone (50 mg/kg, ip). A guide cannula (ICM-23G09; Intermedical, Osaka, Japan) was inserted into lateral ventricle (LV) with the tip located at 0.5 mm caudal, 0.1 mm lateral to the bregma, and 2.2 mm below the skull. Mice were allowed to recover from surgery for at least one week before being subjected to tests. Intraventricular administration of human insulin (100 μU/2 μl) was performed immediately within 30 minutes before the dark period after fasting for 10 hours. At 30 minutes after insulin injection, trunk blood was collected immediately after decapitation without anesthesia. Plasma concentrations of oxytocin (Phoenix Pharmaceutical, Inc, Burlingame, CA) were measured with enzyme immunoassay method according the instruction.

### Immunohistochemistry for pdk1 and oxytocin

Mice were deeply anesthetized with 2,2,2-tribromoethanol and perfused transcardially with 4% paraformaldehyde in 0.1 M phosphate buffer (PB) for 20 min. The brains were removed and subjected to immunohistochemistry. Coronal sections (40 μm) of the hypothalamus were cut using a freezing microtome and collected at 120 μm intervals. The brain was immunostained for PDK1 using rabbit anti-PDK1 antibody (ab52893; Abcam, Cambridge; 1:1000 dilution) and for oxytocin using mouse anti-oxytocin antidody (MAB5296; Millipore, Billerica, MA, 1:1000). Alexa fluor 488 goat anti-rabbit (Life Technologies, Carlsbad, CA; 1:500) and Alexa fluor 594 goat anti-mouse (Life Technologies, Carlsbad, CA; 1:500) were used as the secondary antibodies for PDK1 and oxyotcin, respectively. The confocal fluorescence images for PDK1 and oxyotcin were acquired using Olympus FV1000 confocal laser-scanning microscope (Olympus, Tokyo, Japan).

### Immunohistochemistry for c-Fos and oxytocin

At 90 minutes after LV injection of insulin or vehicle, mouse coronal sections with 40 µm thickness were cut with a freezing microtome and collected at 160 µm intervals. Sections were rinsed in phosphate buffered saline (PBS) and then incubated in 0.3% H_2_O_2_ for 20 min. After rinsing, sections were blocked with 2% bovine serum albumin and 2% normal goat serum for 30 min and incubated with rabbit anti-c-Fos antibody (sc-52, Santa Cruz, 1:5000) overnight at 4 °C. Then the sections were rinsed and incubated with biotinylated goat anti-rabbit IgG for 30 min. After rinsing, sections were incubated with avidin-biotin complex (ABC) reagent for 30 min (Vector Laboratories; 1:500). After rinsing in PBS, color was developed with a nickel-diaminobenzidine (DAB) solution (0.3% nickel ammonium sulfate, 0.02% DAB, and 0.005% H_2_O_2_ in 0.05 M Tris buffer) for 5 min. For double-labeling immunohistochemistry for c-Fos and oxytocin, the process of oxytocin staining was added. After rinsing in PBS, sections were treated with an avidin and biotin blocking solution (Vector Laboratories) and then incubated with mouse anti-oxytocin antibody (Ab2078; Abcam, Cambridge, 1:1000) diluted in a blocking solution overnight at 4 °C. After rinsing, sections were incubated with biotinylated horse anti-mouse IgG antibody for 30 min and incubated in ABC reagent for 30 min. Then the sections were rinsed in PBS and Tris buffer, and color was developed with a DAB solution (0.02% DAB and 0.005% H_2_O_2_ in Tris buffer). Slices were then rinsed, mounted on slides, and coverslipped with Entellan new (Merck, Darmstadt, Germany).

### Measurement of cytosolic calcium concentration ([Ca^2+^]_i_)

Single neurons were isolated from mice aged 5–6 weeks. Briefly, brain slices containing the entire PVN were prepared and the PVN was excised from the left and right sides. The dissected tissues were washed with 10 mM HEPES-buffered Krebs-Ringer bicarbonate buffer (HKRB) containing 1 mM glucose. They were then incubated in 10 mM HKRB containing 1 mM glucose, 20 U/ml papain (Sigma Chemical Co., St. Louis, MO), 0.015 mg/ml deoxyribonuclease, 0.75 mg/ml BSA, and 1 mM cysteine for 15 min at 36 C in a shaking water bath, followed by gentle mechanical trituration for 5–10 min. After trituration, the cell suspension was centrifuged at 100 g for 5 min. The pellet was resuspended in HKRB. The single neurons obtained were distributed onto coverslips and incubated in the humidified chamber at 30 °C for 30 min and then at 25 °C for up to 6 h until use. In previous electrophysiological study of the neurons in paraventricular nucleus, the cells with cellbody >25 μm formed a small population and were considered the magnocellular neurons^17,18^. Therefore, in this study, the single neurons on coverslips with diameter >25 μm were classified as larger-sized neurons, which may largely represent the magnocellular neurons, while those with diameter <25 μm were classified as smaller-sized neurons, which may largely represent the parvocellular neurons. [Ca^2+^]_i_ was measured by ratiometric fura-2 fluorescence imaging. Briefly, single neurons on coverslips were incubated with 2 µmol/l fura-2/AM (Dojin chemical, Kumamoto, Japan) for 40 min at room temperature, mounted in chamber, and superfused with HEPES-buffered Kreb-Ringer bicarbonate buffer (HKRB) composed of (in mM) 129 NaCl, 5.0 NaHCO_3_, 4.7 KCl, 1.2 KH_2_PO_4_, 1.8 CaCl_2_, 1.2 MgSO_4_, 10 HEPES and 5.6 glucose at pH 7.4. The fluorescence images due to excitation at 340 and 380 nm were captured and ratio (F340/F380) images produced by Aquacosmos system (Hamamatsu Photonics, Hamamatsu, Japan). The single neurons subjected to [Ca^2+^]_i_ measurements were subsequently immunostained for oxytocin using rabbit anti-oxytocin antibody (abcam, 1: 1000) as previously described^19^.

### Statistical analysis

Data are expressed as means ± s.e.m. Data were analyzed for statistical significance by one-way ANOVA followed by Tukey’s test. P < 0.05 was considered significant.

## Results

**Figure 1:**
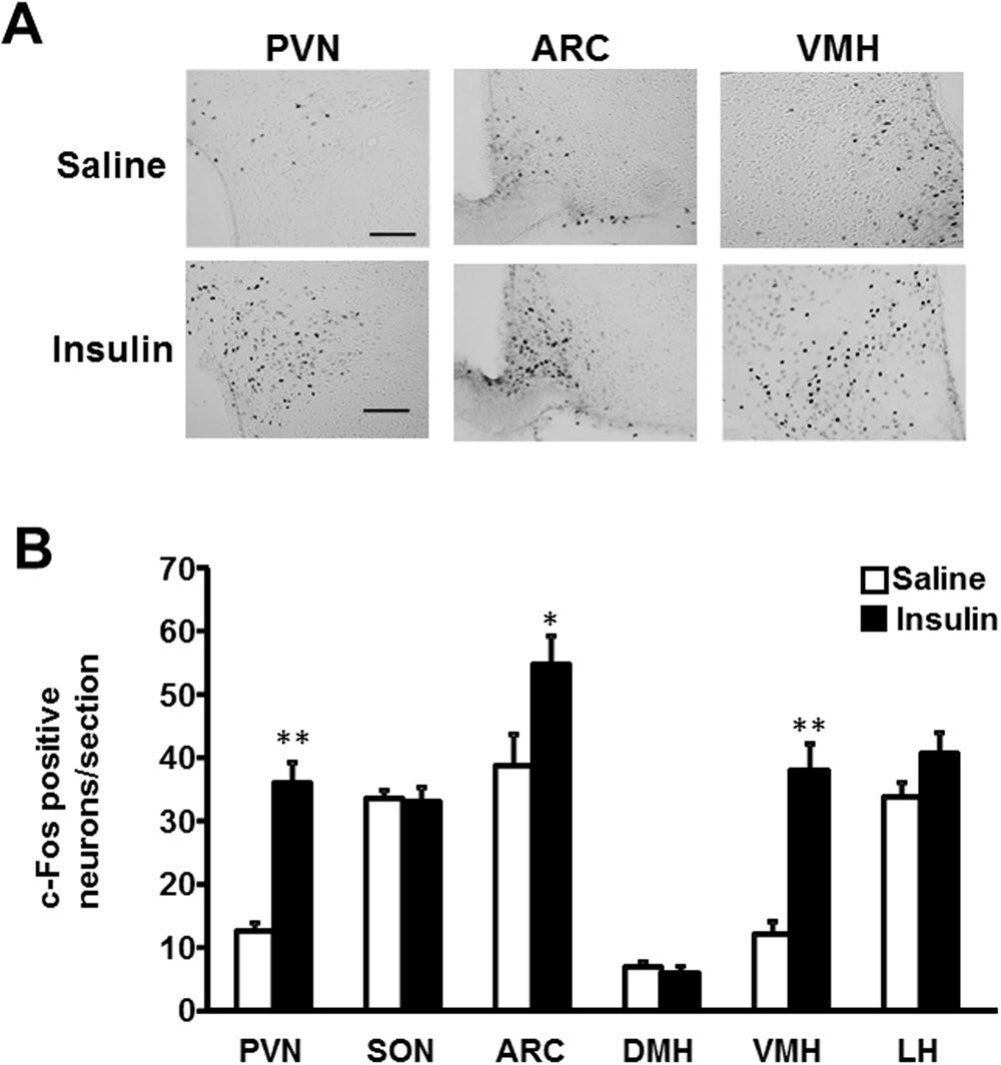
Effect of insulin on c-Fos expression in the hypothalamus. (**A**) Representative pictures showing c-Fos expression in the PVN, ARC and VMH of hypothalamus at 90 min after ICV injection of saline or insulin (100 μU/2 μl). Scale bar: 200 μm. (**B**) Number of c-Fos- immunopositive (IR) neurons per section of the hypothalamic nuclei at 90 min after ICV injection of saline (white bars) or insulin (100 μU/2 μl) (black bars). *n* = 5. **p < 0.01 and *p < 0.05 vs. saline by one-way ANOVA followed by Tukey’s test. Error bars are SEM.

**Figure 2:**
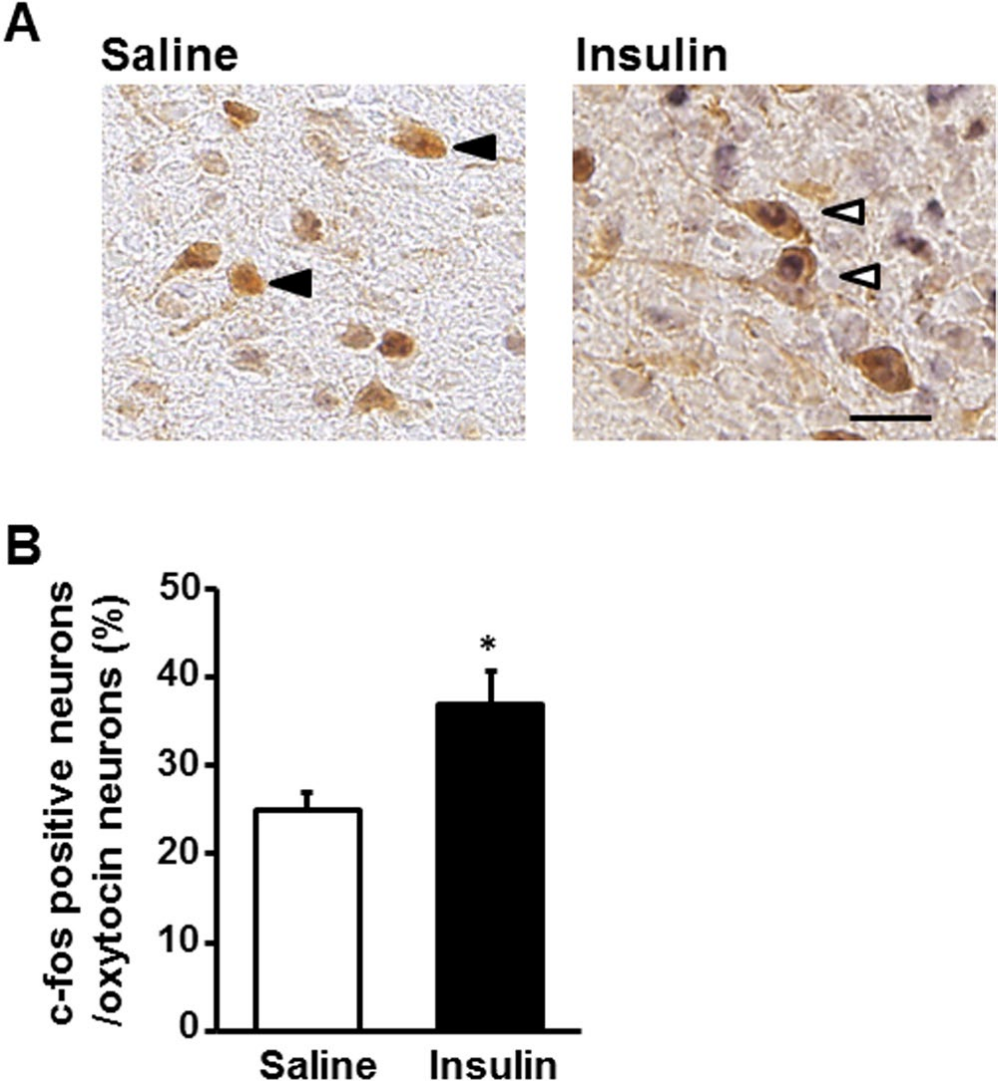
Effect of insulin on c-Fos expression in oxytocin-IR neurons. (**A**) Representative pictures depicting dual immunostaining for c-Fos and oxytocin in PVN after injection of saline or insulin (100 μU/2 μl). Black arrowheads indicate oxytocin-IR neurons. White arrowheads indicate the neurons IR to both oxytocin and c-Fos. Scale bar: 30 μm. (**B**) Incidence of c-Fos-IR neurons in oxytocin-IR neurons. *n* = 5. *p < 0.05 vs. saline by one-way ANOVA followed by Tukey’s test. Error bars are SEM.

**Figure 3:**
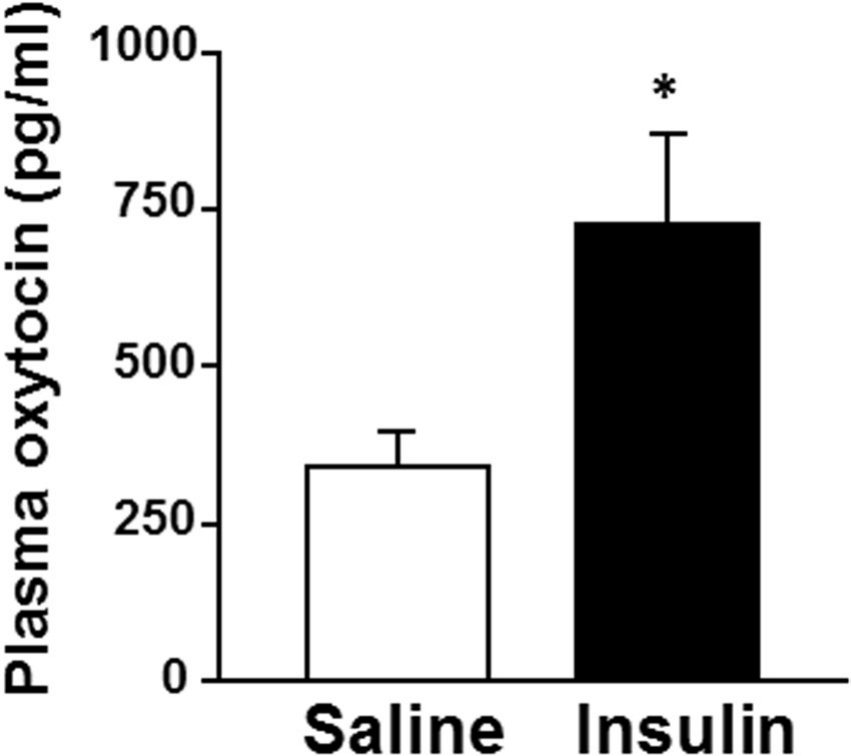
Effect of insulin on plasma oxytocin concentration. Plasma oxytocin concentrations at 30 min after ICV injection of saline or insulin (100 μU/2 μl). *n* = 6–7. *p < 0.05 vs. saline by one-way ANOVA followed by Tukey’s test. Error bars are SEM.

ICV administration of insulin (100 μU), compared to saline, significantly increased c-Fos expression in the PVN, arcuate nucleus (ARC) and ventromedial hypothalamus (VMH), but not SON, in the hypothalamus (Fig. 1A). Next, we performed double-staining for oxytocin and c-Fos in PVN after ICV insulin administration. The c-Fos immunoreactivity was observed in 25% and 38% of oxytocin neurons in PVN after ICV administration of saline and insulin, respectively (Fig. 2). Thus, insulin increased the incidence of the oxytocin neurons immunoreactive (IR) to c-Fos approximately by 13%. Furthermore, ICV insulin administration doubled the plasma oxytocin concentration (Fig. 3). These results indicated that insulin activates PVN oxytocin neurons *in vivo*.

**Figure 4:**
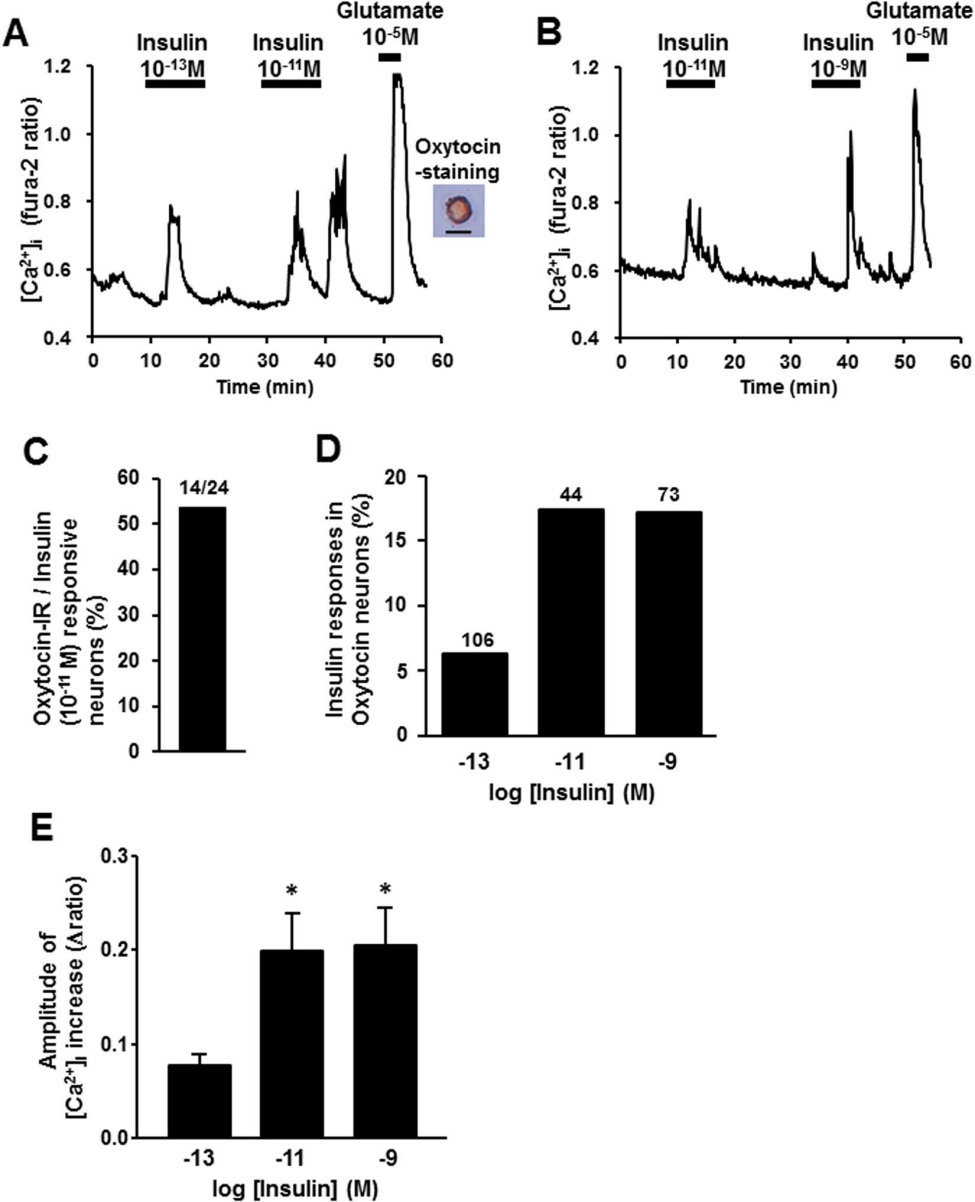
Insulin increases [Ca^2+^]_i_ in single PVN oxytocin neurons. (**A**) Insulin at 10^−13^ M and 10^−11^ M increased [Ca^2+^]_i_ (left panel) in a single PVN neuron that was subsequently shown to be IR to oxytocin (right panel). Scale bar is 25 μm. (**B**) Insulin at 10^−11^ and 10^−9^ M increased [Ca^2+^]_i_ in a single PVN neuron. (**C**) 14 of 24 (53.8%) neurons that responded to insulin were IR to oxytocin. (**D**) Incidence of [Ca^2+^]_i_ responses to insulin in PVN oxytocin neurons, expressed by percentage. Numbers around each point indicate the number of PVN oxy neurons. (**E**) Average amplitude of insulin-induced [Ca^2+^]_i_ increases (Δratio) in oxytocin neurons. *p < 0.05 vs. basal level by one-way ANOVA followed by Tukey’s test. Error bars are SEM.

To investigate whether insulin activates PVN oxytocin neurons by a direct action, we measured cytosolic calcium concentration ([Ca^2+^]_i_) in the single neurons isolated from PVN, followed by immunocytochemical identification of oxytocin neurons. Administration of insulin at 10^−13^ M, 10^−11^ M and 10^−9^ M increased [Ca^2+^]_i_ in the PVN neurons that were subsequently shown to be IR to oxytocin (Fig. 4A and B). Insulin at 10^−13^ M increased [Ca^2+^]_i_ with small amplitude in 6% of oxytocin-IR PVN neurons, and at 10^−11^ M and at 10^−9^ M it increased [Ca^2+^]_i_ with larger amplitude in 18% of oxytocin-IR PVN neurons (Fig. 4D and E). Thus, insulin at 10^−11^ M showed the maximal effect to increase [Ca^2+^]_i_ in PVN oxytocin neurons. Furthermore, 14 of 24 insulin (10^−11^ M)-responsive PVN neurons (53.8%) were IR to oxytocin (Fig. 4C), indicating that the oxytocin neuron is a major target for insulin in PVN. These results revealed that insulin directly activated oxytocin neurons and insulin action was maximal at 10^−11^ M.

**Figure 5:**
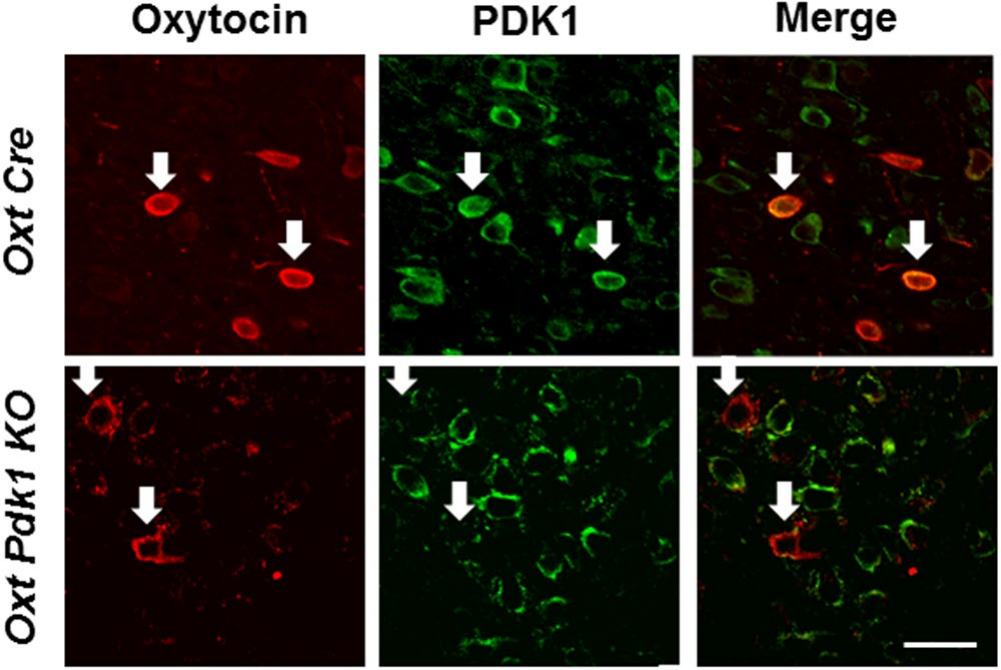
Double immunostaining for oxytocin and PDK1 in PVN of *Oxy Cre* and *Oxy Pdk1* KO mice. Double immunostaining for oxytocin and PDK1 in PVN. The upper rows show *Oxy Cre* mice and the lower rows *Oxy Pdk1* KO mice. Left: oxytocin (Red: Alexa 594 fluorescence), middle: PDK1 (Green: Alexa 488), right: merged images. Scale bar: 30 μm. White arrows: oxytocin-IR neurons.

**Figure 6:**
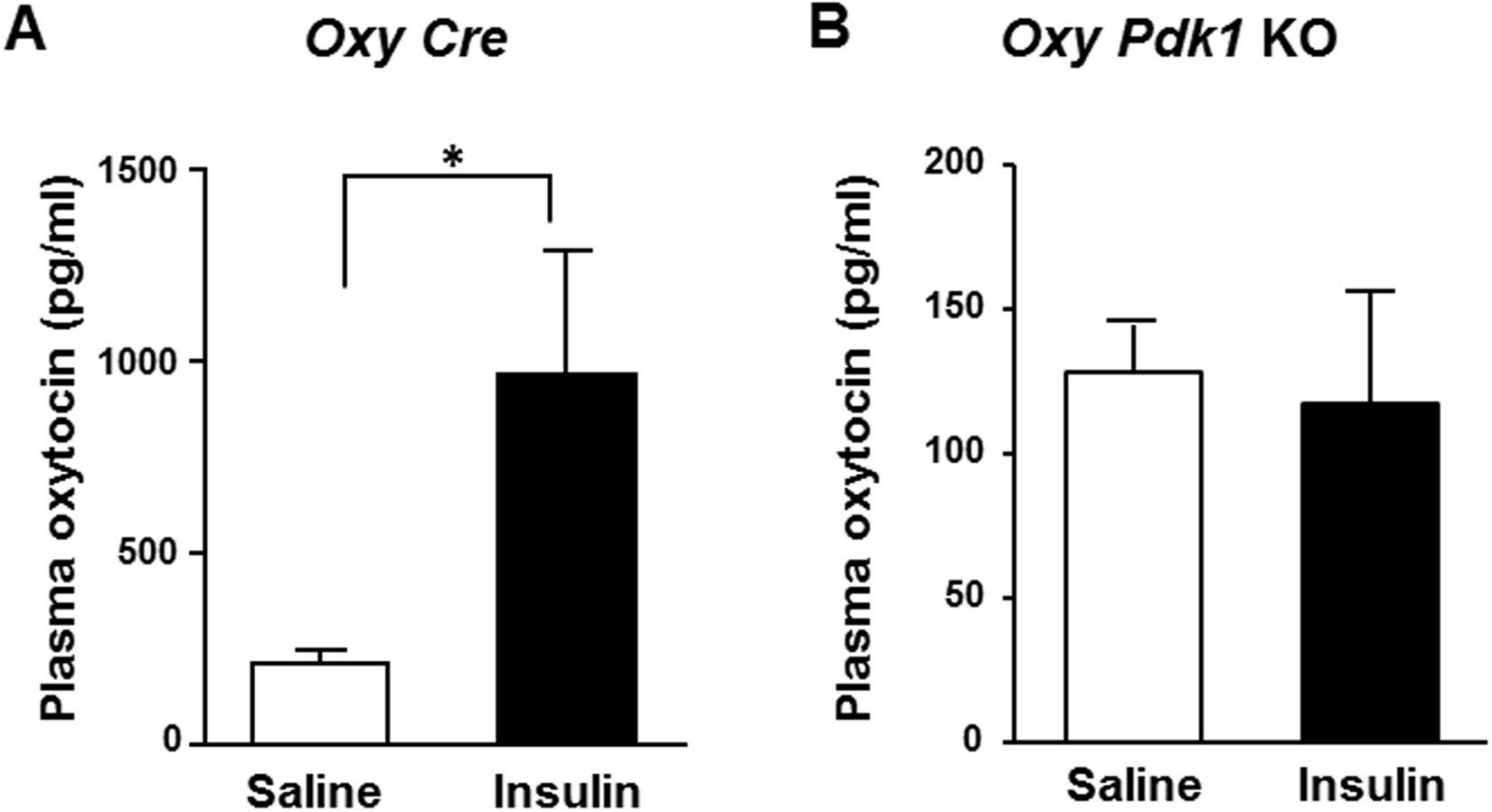
Insulin fails to increase plasma oxytocin concentrations in *Oxy Pdk1* KO mice. Plasma oxytocin concentrations at 30 min after ICV injection of saline or insulin (100 μU/2 μl) in *Oxy Cre* mice (**A**) and *Oxy Pdk1* KO mice (**B**). n = 6. *p < 0.05 vs. saline by one-way ANOVA followed by Tukey’s test. Error bars are SEM.

Activation of insulin receptors phosphorylates the insulin receptor substrate (IRS) proteins, which in turn activate phosphatidylinositol-3 kinase (PI3K), an enzyme that generates phosphatidylinositol-3, 4, 5-trisphosphate (PIP3). PIP3 activates 3-phosphoinositide-dependent protein kinase 1 (PDK1), which in turn activates protein kinase B (PKB, also known as Akt) and members of the atypical PKC family^20^. To investigate the role of PDK1 in the action of insulin in PVN oxytocin neurons, we generated the oxytocin neuron specific PDK1 knockout (*Oxy Pdk1* KO) mice. In the control *Oxy Cre* mice, expression of immunofluorescence for PDK1 was observed in oxytocin neurons in PVN, but absent in those neurons in *Oxy Pdk1* KO mice (Fig. 5). In *Oxy Cre* mice, ICV injection of insulin (100 μU), compared to saline, markedly elevated the plasma oxytocin concentration at 30 minutes after injection (Fig. 6A). In *Oxy Pdk1* KO mice, in contrast, ICV injection of insulin failed to significantly alter plasma oxytocin at the same time point (Fig. 6B). These results suggest that the PDK1 in oxytocin neurons mediates the insulin action to stimulate oxytocin release.

**Figure 7:**
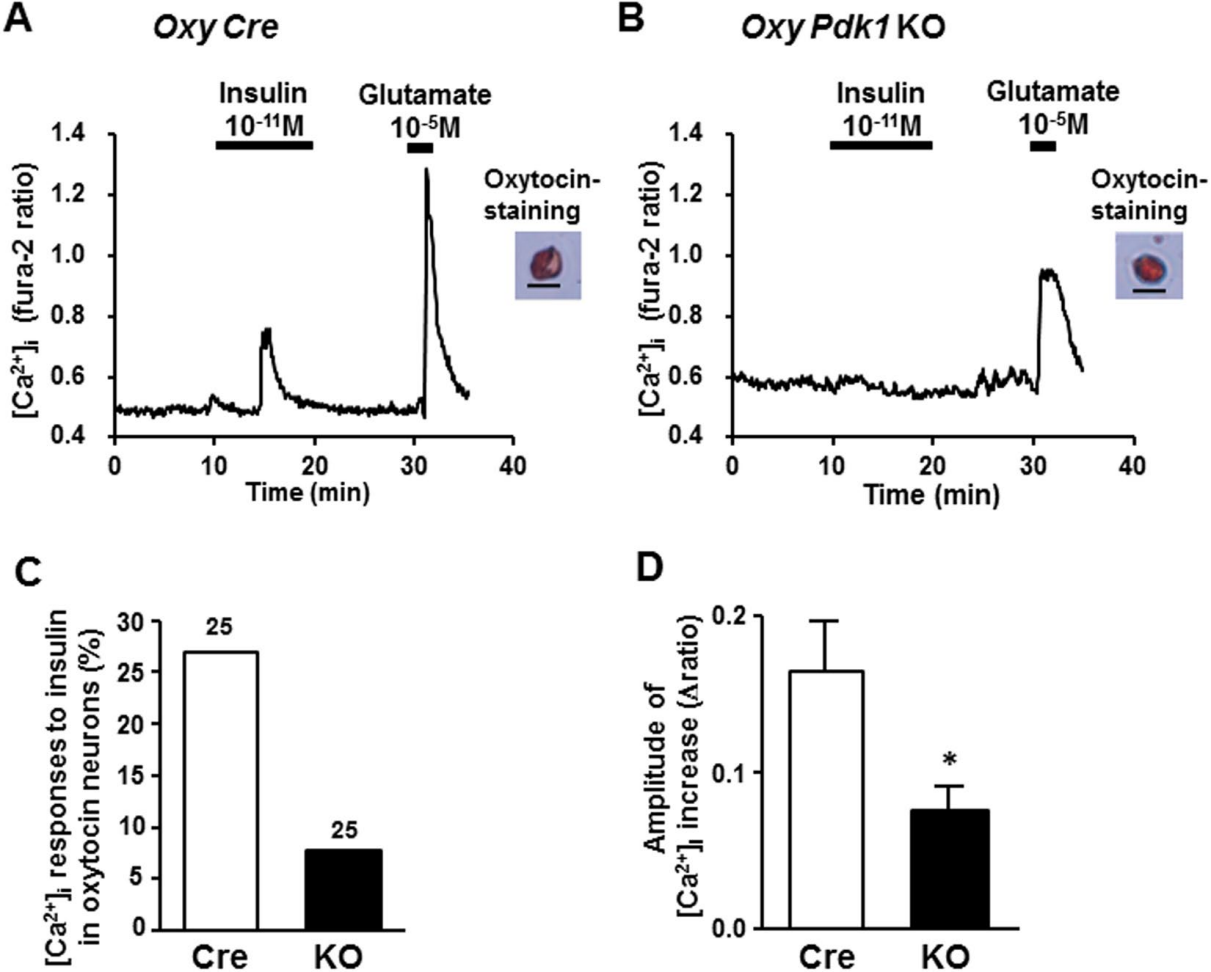
Insulin fails to substantially increase [Ca^2+^]_i_ in larger-sized oxytocin neurons of PVN in *Oxy Pdk1* KO mice. (**A**,**B**) Representative [Ca^2+^]_i_ responses to 10^−11^ M insulin in larger-sized oxytocin neurons of PVN, possibly representing magnocellular neurons, isolated from *Oxy Cre* mice (**A**) and *Oxy Pdk1* KO mice (**B**). Single neurons with diameter >25.0 μm were classified as larger-sized neurons in this study. These neurons from both mice similarly responded to 10^−5^ M glutamate. (**C**) Incidence of [Ca^2+^]_i_ responses to insulin in PVN oxytocin neurons, expressed by percentage. Numbers around each point indicate the number of PVN oxy neurons. (**D**) Average amplitude of [Ca^2+^]_i_ responses to insulin (Δratio) in oxytocin neurons. Scale bar: 25 μm. *p < 0.05 vs. *Oxy Cre* mice by one-way ANOVA followed by Tukey’s test. Error bars are SEM.

**Figure 8:**
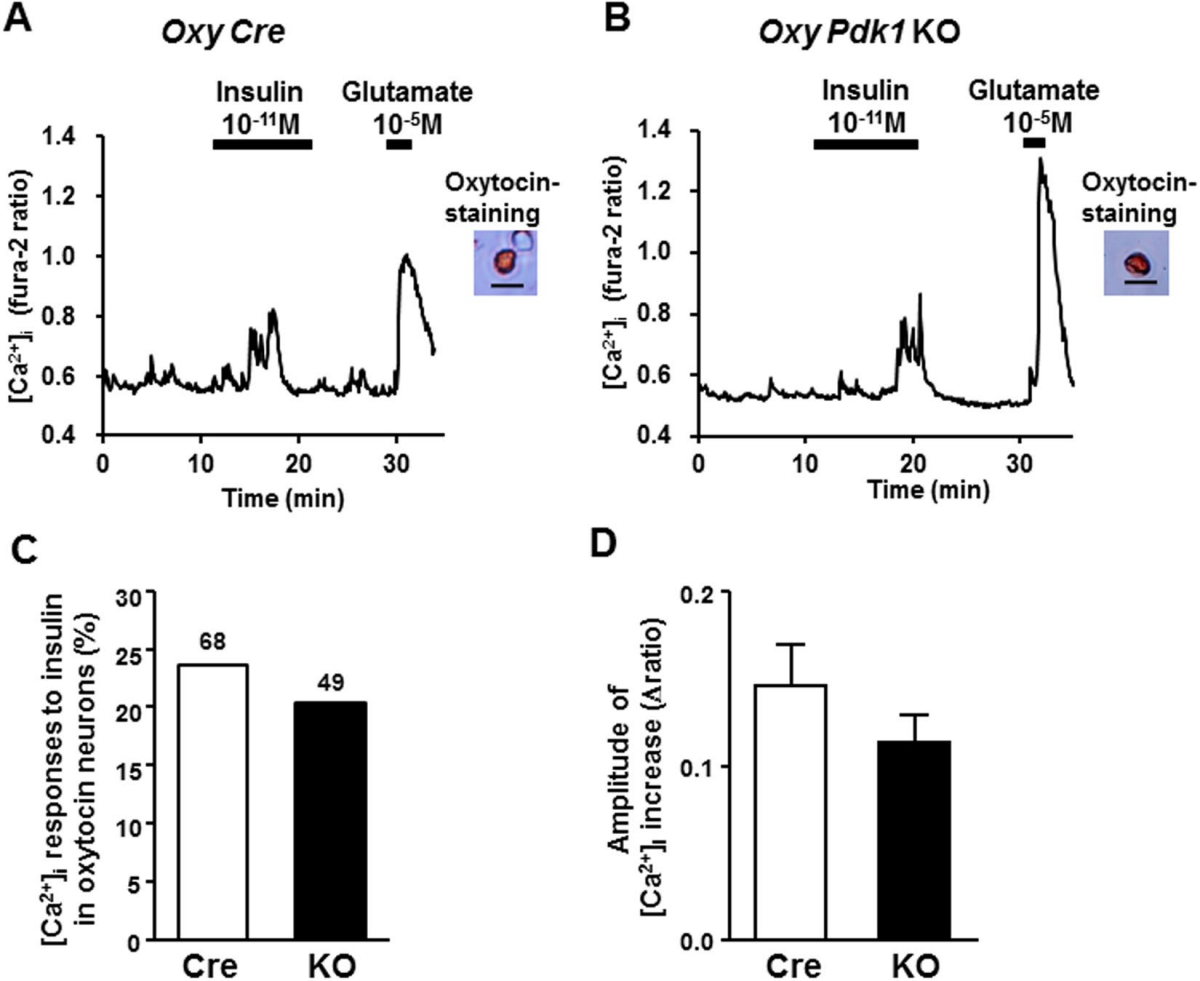
Insulin increases [Ca^2+^]_i_ in smaller-sized oxytocin neurons of PVN. (**A**,**B**) Representative [Ca^2+^]_i_ responses to 10^−11^ M insulin in smaller-sized oxytocin neurons of PVN, possibly representing parvocellular neurons, isolated from *Oxy Cre* mice (**A**) and *Oxy Pdk1* KO mice (**B**). Single neurons with diameter <25.0 μm were classified as smaller-sized neurons in this study. (**C**) Incidence of [Ca^2+^]_i_ responses to insulin in PVN oxytocin neurons, expressed by percentage. Numbers around each point indicate the number of PVN oxy neurons. (**D**) Average amplitude of [Ca^2+^]_i_ responses to insulin (Δratio) in oxytocin neurons examined. Scale bar: 25 μm.

We next examined whether insulin directly activates the larger-sized oxytocin neurons in PVN, which may represent magnocellular neurons, and if so, this action depends on PDK1. For this, we measured [Ca^2+^]_i_ in single oxytocin neurons isolated from PVN of *Oxy Cre* mice and *Oxy Pdk1* KO mice. The oxytocin neurons with diameter of 25 μm or larger were defined as larger-sized neurons. Insulin at 10^−11^ M interacted with and increased [Ca^2+^]_i_ in the larger-sized oxytocin neurons of *Oxy Cre* mice (Fig. 7A,C,D). The [Ca^2+^]_i_ increase with substantial amplitude occurred in about 27% of larger-sized oxytocin neurons from *Oxy Cre* mice. In contrast, 10^−11^ M insulin increased [Ca^2+^]_i_ only in 7% of those neurons frorm *Oxy Pdk1* KO mice (Fig. 7B,C,D). Thus, the ability of insulin to increase [Ca^2+^]_i_ in larger-sized oxytocin neurons was markedly diminished in *Oxy Pdk1* KO mice (Fig. 7A–D). These results indicated that insulin activates the larger-sized oxytocin neuron of PVN via the signaling pathway involving Pdk1. In contrast, the smaller-sized (<25 μm) parvocellular oxytocin neurons from *Oxy Cre* mice and those from *Oxy Pdk1* KO mice equally responded to insulin (Fig. 8A and B): both incidence and amplitude of [Ca^2+^]_i_ increases in smaller-sized neurons in response to insulin were not significantly different between *Oxy Cre* and *Oxy Pdk1* KO mice (Fig. 8C and D).

## Discussion

In this study, we found that insulin directly interacts with the larger-sized PVN oxytocin neurons to increase [Ca^2+^]_i_ via signaling involving PDK1 and that this neuronal activation is relayed to oxytocin release in circulation. The present study has identified the PVN oxytocin neuron as a novel and substantial target for the central action of insulin.

In the brain as well as peripheral tissues, PDK1, the downstream of PI 3-kinase, serves as a key molecule in insulin signaling cascade. It has previously been shown that PDK1 is implicated in the [Ca^2+^]_i_ responses to various stimuli in both AgRP/NPY and POMC neurons in the arcuate nucleus (ARC)^21–23^. We here showed that the insulin action to promote the activity of PVN larger-sized oxytocin neurons and oxytocin release were blunted in *Oxy Pdk1* KO mice. These results reveal a critical role of PDK1 in the PVN larger-sized oxytocin neurons, which couples the central insulin action to the neuronal activity and release of oxytocin. It has recently been reported that insulin promotes oxytocin release from SON oxytocin neurons via PI-3 kinase signaling^24^. This previous and our current studies taken together suggest that insulin induces oxytocin release through the PI 3-kinase-PDK1 pathway.

Although insulin can pass through the blood brain barrier, the insulin concentration in the cerebrospinal fluid is reportedly much lower than that in the plasma, producing a large concentration difference greater than 10 times in humans^25,26^. Transportation of insulin to the cerebrospinal fluid is slow, and little influenced by dietary property in humans^27,28^. The insulin concentration in human cerebrospinal fluid at fasting condition is <10 pmol/L (10^−11^ M), a level that is too low to activate the insulin receptor in peripheral tissues. Surprisingly, from the analysis of isolated neurons in the current study, the PVN oxytocin neurons are activated by insulin at the concentrations as low as 10^−13^ to 10^−11^ M. This finding supports that the PVN oxytocin neurons can be activated by the low levels of insulin present in cerebrospinal fluid. The mechanism that renders the PVN oxytocin neurons responsive to these low concentrations of insulin remains to be elucidated. However, the sensitivity of single PVN oxytocin neurons to insulin could be increased by 5 mM glucose present in superfusion solution in the current [Ca^2+^]_i_ measurement. It was reported that the SON oxytocin neuron is activated by co-administration of glucose (5 mM) and insulin. We previously reported that insulin and glucose synergistically increased [Ca^2+^]_i_ in PVN nesfatin-1 neurons^29^. Therefore, oxytocin neurons, when primed with high glucose concentration, may be activated by the levels of insulin present in cerebrospinal fluid after meal.

The plasma concentrations of oxytocin are significantly decreased in obese and diabetic patients^16,30–32^. Moreover, expression and plasma concentration of oxytocin also decreased in obese model animals^14,30,32^. The insulin resistance in the brain as well as peripheral tissues occurs in obese subjects and animals^32–35^. The present finding that ICV injection of insulin promotes oxytocin secretion suggest that insulin resistance in oxytocin neurons could serve as a factor that leads to the reduction in oxytocin concentrations in obese and/or type 2 diabetic patients.

Recently oxytocin, besides its physiological function in parturition and lactation, also regulate appetite and metabolism^36–39^. Intranasal administration of oxytocin in humans has been reported to ameliorate overeating^39–43^ and ameliorates hyperglycemia^44^. Moreover, ICV injection of oxytocin decreases food intake in both normal-weight and obese animals^14,45^. Furthermore, oxytocin enhances energy expenditure, a centrally-regulated function^46^. Thus, the beneficial effects of oxytocin on body weight regulation largely depend on the central action. In the present study, insulin activated the PVN parvocellular, as well as magnocellular, oxytocin neurons that project to several brain regions. It was suggested that PVN parvocellular oxytocin neurons play a role in central action of insulin to regulate energy metabolism. We furthermore found that insulin increases [Ca^2+^]_i_ in the PVN smaller-sized oxytocin neurons, which may largely represent their parvocellular subpopulation, in a PDK1-independent manner, being contrasted to the PDK1-dependent action in the larger-sized magnocellular oxytocin neurons. This finding suggests the signaling pathway-dependent sorting of insulin-induced multiple oxytocin functions. Further studies are required to further elucidate the insulin signaling mechanisms in the PVN parvocellular and magnocellular oxytocin neurons and their link to specific functions, which could help utilize a specific branch of the insulin-oxytocin axis for treating obesity, diabetes and/or perinatal disorders.
